# Single-electron operations in a foundry-fabricated array of quantum dots

**DOI:** 10.1038/s41467-020-20280-3

**Published:** 2020-12-16

**Authors:** Fabio Ansaloni, Anasua Chatterjee, Heorhii Bohuslavskyi, Benoit Bertrand, Louis Hutin, Maud Vinet, Ferdinand Kuemmeth

**Affiliations:** 1 Center for Quantum Devices, Niels Bohr Institute, University of Copenhagen, 2100 Copenhagen, Denmark; 2 CEA, LETI, Minatec Campus, Grenoble, France

**Keywords:** Quantum information, Quantum dots

## Abstract

Silicon quantum dots are attractive for the implementation of large spin-based quantum processors in part due to prospects of industrial foundry fabrication. However, the large effective mass associated with electrons in silicon traditionally limits single-electron operations to devices fabricated in customized academic clean rooms. Here, we demonstrate single-electron occupations in all four quantum dots of a 2 x 2 split-gate silicon device fabricated entirely by 300-mm-wafer foundry processes. By applying gate-voltage pulses while performing high-frequency reflectometry off one gate electrode, we perform single-electron operations within the array that demonstrate single-shot detection of electron tunneling and an overall adjustability of tunneling times by a global top gate electrode. Lastly, we use the two-dimensional aspect of the quantum dot array to exchange two electrons by spatial permutation, which may find applications in permutation-based quantum algorithms.

## Abstract (web summary)

Semiconductor spin-qubits with CMOS compatible architectures could benefit from the industrial capacity of the semiconductor industry. Here, the authors make the first steps in demonstrating this by showing single electron operations within a two-dimensional array of foundry-fabricated quantum dots.

## Introduction

Silicon spin qubits have achieved high-fidelity one- and two-qubit gates^1–5^, above error-correction thresholds^6^, promising an industrial route to fault-tolerant quantum computation. A significant next step for the development of scalable multi-qubit processors is the operation of foundry-fabricated, extendable two-dimensional (2D) quantum-dot arrays. In gallium arsenide, 2D arrays recently allowed coherent spin operations and quantum simulations^7,8^. In silicon, 2D arrays have been limited to transport measurements in the many-electron regime^9^.

Here, we operate a foundry-fabricated 2 × 2 array of silicon quantum dots in the few-electron regime, achieving single-electron occupation in each of the four gate-defined dots, as well as reconfigurable single, double, and triple dots with tunable tunnel couplings. Pulsed-gate and gate-reflectometry techniques permit single-electron manipulation and single-shot charge readout, while the two-dimensionality allows the spatial exchange of electron pairs. The compact form factor of such arrays, whose foundry fabrication can be extended to larger 2 × *N* arrays, along with the recent demonstration of spin control^10–12^ and spin readout^13,14^, paves the way for dense qubit arrays for quantum computation and simulation^15^.

## Results

### Device and gate reflectometry

**Fig. 1:**
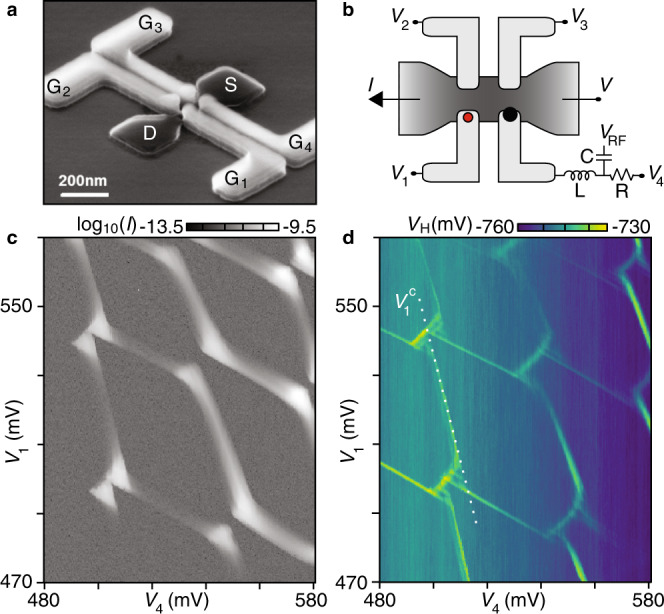
Compensated control voltages within a two-dimensional silicon quantum-dot array. **a** Foundry-fabricated undoped silicon channel connected to reservoirs (dark gray), with four gate electrodes (light gray). This SEM image shows a device from a different fabrication run without backend^16^. **b** Device schematic for the example of a few-electron double dot underneath gates G_1_ and G_4_, induced by appropriate control voltages *V*_1–4_. Each of the three qubit dots (dot 1 indicated in red) capacitively couples to the sensor dot (black), which can be monitored using RF reflectometry off an inductor (L) wirebonded to G_4_. **c**, **d** Charge stability diagram of the double quantum dot in **b**, acquired at fixed source–drain bias *V* = −3 mV. Source–drain current *I* and demodulated reflectometry voltage *V*_H_ measured simultaneously as a function of *V*_1_ and *V*_4_. The dotted white line defines a compensated voltage ${V}_{{\rm{1}}}^{\mathrm{c}}$ that controls the chemical potential of dot 1 without affecting the chemical potential of dot 4. Control voltages ${V}_{{\rm{1,2,3}}}^{\mathrm{c}}$ for other dot configurations are established analogously.

Our device architecture consists of an undoped silicon channel (Fig. 1a, dark gray) connected to a highly doped source (S) and drain (D) reservoir. Metallic polysilicon gates (light gray) partially overlap the channel, each capable of inducing one quantum dot with a controllable number of electrons^16,17^.

While devices with a larger number of split-gate pairs are possible (see Supplementary Fig. S1 and refs. ^17,18^), we focus on a 2 × 2 quantum-dot array as the smallest two-dimensional unit cell in this architecture, that is, a device with two pairs of split-gate electrodes, labeled G_*i*_ with corresponding control voltages *V*_*i*_. The device studied is similar to the one shown in Fig. 1a, but additionally has a common top gate 300 nm above the channel, and was encapsulated at the foundry by a backend that includes routing to wirebonding pads. Quantum dots are induced in the 7-nm-thick channel by 32-nm-long gates, separated from each other by 32-nm silicon nitride (see “Methods”). The handle of the silicon-on-insulator wafer is grounded during measurements, but can in principle be utilized as a back gate. Figure 1b shows a schematic of the device with *V*_*i*_ tuned to induce a few-electron double quantum dot underneath G_1_ and G_4_. Source and drain contacts allow conventional *I*(*V*) transport characterization, while an inductor (wirebonded to G_4_) allows gate-based reflectometry, in which the combination of a radio-frequency (RF) carrier (*V*_RF_) and a homodyne detection circuit yields a demodulated voltage *V*_H_^19^. Bias tees connected to G_1−3_ (not shown) allow the application of high-bandwidth voltage signals.

Measurement of the source–drain current *I* as a function of *V*_1_ and *V*_4_ reveals a conventional double-dot stability diagram (Fig. 1c), with bias triangles arising from a finite source bias *V* = −3 mV and co-tunneling ridges indicating substantial tunnel couplings in this few-electron regime (each dot is occupied by 6–9 electrons). The characteristic honeycomb pattern is also observed in the demodulated voltage *V*_H_ (Fig. 1d, acquired simultaneously with Fig. 1c), and suggests the potential use of G_4_ for (dispersively) sensing charge rearrangements (quantum capacitance) anywhere within the 2D array. In the following, we keep dot 4 in the few-electron regime (6–9 electrons, serving as a sensor dot), resulting in an enhancement of *V*_H_ whenever dot 4 exchanges electrons with its reservoir, and reduce the occupation numbers of the other three dots (which in the single-electron regime we refer to as qubit dots). In fact, the large capacitive shift of the dot-4 transition by nearby electrons (evident in Fig. 1c for dot 1) was used to count the absolute number of electrons within each of the three qubit dots (see “Methods”).

### Single-electron control

It is convenient to control the chemical potential of the three qubit dots without affecting the chemical potential of the sensor dot, as illustrated for dot 1 by the compensated control parameter ${V}_{{\mathrm{1}}}^{{\mathrm{c}}}$ (Fig. 1d). This is done experimentally by calibrating the capacitive matrix elements *α*_*i*4_ such that *V*_4_ compensates for electrostatic cross-coupling between *V*_1−3_ and dot 4, that is, by updating voltage ${V}_{{\rm{4}}}={V}_{{\rm{4}}}^{{\mathrm{o}}}-\mathop{\sum }\nolimits_{{{i}} = 1}^{3}{\alpha }_{{{4i}}}({V}_{{{i}}}-{V}_{{{i}}}^{{\mathrm{o}}})$ whenever *V*_1−3_ is changed relative to a chosen reference point $({{V}_{1}^{\mathrm{o}}},{{V}_{2}^{\mathrm{o}}},{{V}_{3}^{\mathrm{o}}})$. The presence of this compensation is indicated by adding a superscript “c” to the respective control parameters. Using this compensation, and setting the operating point of dot 4 with ${{V}_{4}^{\mathrm{o}}}$, the associated reflectometry signal *V*_H_ can be used to detect charge movements between the three qubit dots.

**Fig. 2:**
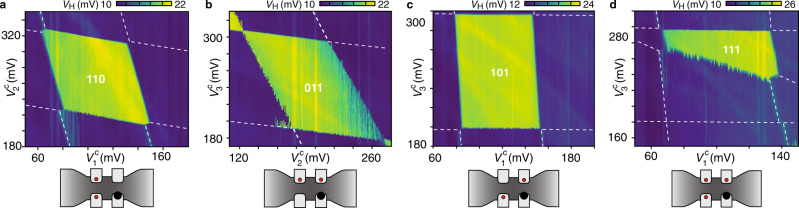
Various single-electron configurations within the array. **a**–**c** Three different double-dot configurations, controlled by compensated voltages ${V}_{{{1,2,3}}}^{\mathrm{c}}$. Numbers indicate the occupation of the three qubit dots (each red dot represents one electron). **d** Similar to **c**, but with ${V}_{{{2}}}^{\mathrm{c}}$ fixed at a larger positive voltage, revealing the triple-dot ground-state region. In **a**–**d** the top gate is fixed at 6 V.

The compensated voltages are used to map out ground-state regions of various desired charge configurations of the qubit dots. For example, Fig. 2a was acquired by first parking *V*_1_ and *V*_2_ in the first Coulomb valley of dot 1 and dot 2 (keeping dot 3 empty by setting *V*_3_ = 0), then tuning *V*_4_ to the degeneracy point of dot 4 (maximum of *V*_H_), before sweeping ${V}_{{\mathrm{2}}}^{{\mathrm{c}}}$ vs. ${V}_{{\mathrm{1}}}^{{\mathrm{c}}}$. The enhancement of *V*_H_ clearly shows the extent of the 110 ground-state region. (Here, numbers indicate the occupation of the three qubit dots, as illustrated in the schematics of Fig. 2.) Due to the relatively large capacitive coupling of the sensor dot to the qubit dots, dot 4 is in Coulomb blockade outside the 110 region; there *V*_H_ reduces to its approximately constant background. (The gain of the reflectometry circuit had been changed relative to the acquisition in Fig. 1d.)

In addition to the transverse double dot in Fig. 2a, we also demonstrate the longitudinal (Fig. 2b, with *V*_1_ = 0) and diagonal (Fig. 2c, with *V*_2_ = 0) double dots. While such a degree of single-electron charge control is impressive for a reconfigurable, silicon-based multi-dot circuit, it is not obvious how coherent single-spin control (e.g., via micromagnetic field gradients^20^ or spin–orbit coupling^12^) can most easily be implemented in these foundry-fabricated structures. One option is to encode qubits in suitable spin states of 111 triple dots, and operate these as voltage-controlled exchange-only qubits^21,22^. To this end, we demonstrate in Fig. 2d the tune-up of a triple dot (in order to populate also dot 2, *V*_2_ = 197 mV was chosen more positive relative to Fig. 2c), revealing the pentagonal cross-section expected for the 111 charge state.

### Tuning of tunneling times

**Fig. 3:**
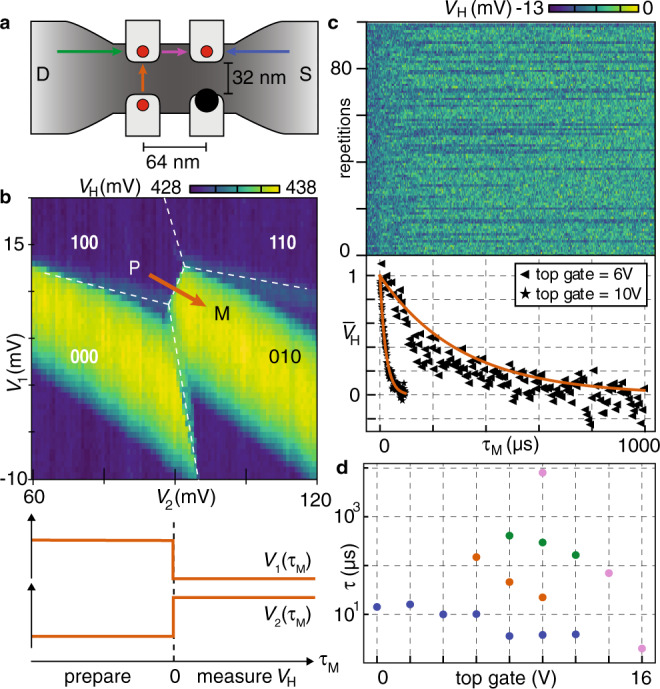
Pulsed-gate charge manipulation, single-shot readout, and tunability of tunnel couplings. **a** Device schematic indicating the lead-to-dot (green and blue) and interdot (orange and magenta) transitions for the first electron. The arrows indicate the directions of the tunneling events studied. **b** Illustration of a *V*_1_–*V*_2_ gate-voltage pulse (orange) that moves an electron from dot 1 to dot 2, with *V*_4_ fixed such that a tunneling event causes a change in the sensor signal *V*_H_ (color scale). For each pulse, digitization of *V*_H_(*τ*_M_) begins when the gate-voltage switches from preparation point P to measurement point M. **c** Single-shot traces *V*_H_(*τ*_M_) for 100 pulse repetitions, with top gate fixed at 6 V. An exponential decay (orange), fitted to the normalized average of all traces (◂), yields a characteristic tunneling time of 300 μs, and is compared with data obtained with the top gate fixed at 10 V (⋆). Analogous data for the other transitions in **a** are shown in Supplementary Fig. S2. **d** Tunneling times for the transitions indicated in **a**, as a function of the top-gate voltage.

To demonstrate fast single-shot charge readout of the qubit dots, we apply voltage pulses to G_1_–G_3_ while digitizing *V*_H_^19^. Specifically, two-level voltage pulses *V*_1,2,3_(*t*) are designed to induce one-electron tunneling events into the quantum-dot array or within the array, as illustrated by color-coded arrows in Fig. 3a. One such pulse is exemplified in Fig. 3b, preparing one electron in dot 1 (P) before moving it to dot 2 for measurement (M). P and M are chosen such that the ground-state transition of interest (in this case the interdot transition) is expected halfway between P and M, using a pulse amplitude of 2 mV. This pulse is repeated many times, with *V*_4_ fixed at a voltage that gives good visibility of the transition of interest in *V*_H_(*τ*_M_). Here, *V*_H_(*τ*_M_) serves as a single-shot readout trace that probes for a tunneling event at time *τ*_M_ after the gate voltages are pulsed to the measurement point (Fig. 3).

Figure 3c shows the repetition of 100 such readout traces obtained at a top-gate voltage of 6 V, revealing the stochastic nature of tunneling events, in this case with an averaged tunneling time of 300 μs. This time is obtained by averaging all single-shot traces and fitting an exponential decay. In the lower panel of Fig. 3c, ${\bar{V}}_{{\rm{H}}}$ indicates that the average (triangles) has been normalized according to the offset and amplitude fit parameters, which allows comparison with similar data (stars) obtained at a top-gate voltage of 10 V (see “Methods”). The deviation of the data from the fitted exponential decay (solid line) may indicate the presence of multiple relaxation processes, and the reported decay times should therefore be understood as an approximate quantification of characteristic tunneling times within the array.

While the compact one-gate-per-qubit architecture in accurately dimensioned silicon devices^10,12^ may ultimately facilitate the wiring fanout of a large-scale quantum computer^23^, an overall tunability of certain array parameters may initially be essential. Figure 3d demonstrates phenomenologically that all transition times studied can be decreased significantly by increasing the top-gate voltage. (The specific gate voltages associated with each data point are listed in Supplementary Table S1.)

### Electron shuttling in two dimensions

An important resource for tunnel-coupled two-dimensional qubit arrays is the ability to move or even exchange individual electrons (and their associated spin states) in real space^24^. In fact, a two-dimensional triple dot, as in our device, is the smallest array that allows the exchange of two isolated electrons (Heisenberg spin exchange, as demonstrated in linear arrays^25^, requires precisely timed wavefunction overlap).

**Fig. 4:**
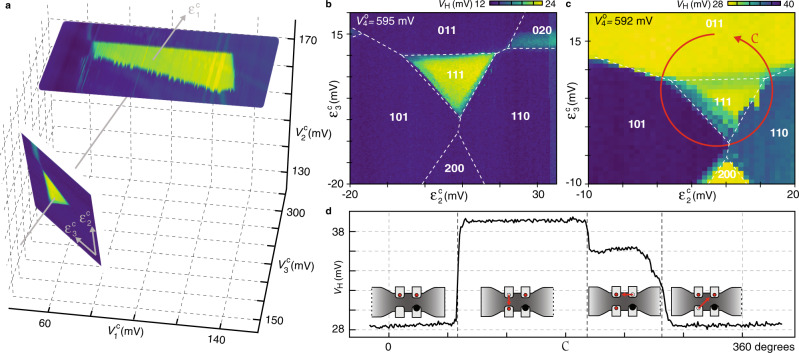
Exchange of two electrons by permutation within a 2D array. **a** Ground-state region of the 111 triple dot from Fig. 2d (${V}_{{{2}}}^{\mathrm{c}}=$constant), plotted in three-dimensional control-voltage space, along measurements within a plane of fixed common-mode voltage (${\epsilon }_{{{1}}}^{\mathrm{c}}=$constant). Physically, ${\epsilon }_{{{1}}}^{\mathrm{c}}$ induces overall gate charge in the array, whereas detuning ${\epsilon }_{{{2}}}^{\mathrm{c}}$ (${\epsilon }_{{{3}}}^{\mathrm{c}}$) relocates gate charge within the array along (across) the silicon channel. **b** Guides to the eye indicating different ground states within the detuning plane in **a**. For this choice of sensor operating point, ${{V}_{4}^{\mathrm{o}}}=595\,$ mV, *V*_H_ does not discriminate between different two-electron configurations. **c** Same detuning plane as in **b**, but with slightly different sensor operating point, ${{V}_{4}^{\mathrm{o}}}=592\,$ mV. The control-voltage path C traverses three two-electron ground states in such a way that the two isolated electrons are exchanged within the array. **d** Sensor signal *V*_H_ acquired during one cycle of the shuttling path C. Changes in *V*_H_ reflect single-electron movements within the array, as illustrated by red arrows. After completion of one cycle C, the position of the two electrons in the array has been permuted.

To demonstrate the spatial exchange of two electrons, we first follow the 111 ground-state region of Fig. 2d towards lower voltages on G_1−3_. In Fig. 4a, this is accomplished by reducing the common-mode voltage ${\epsilon }_{{\rm{1}}}^{{\rm{c}}}$, such that the 111 region only borders with two-electron ground states. In this gate-voltage region, the charge configuration of the qubit dots is most intuitively controlled using a symmetry-adopted coordinate system defined by

1 $$\left(\begin{array}{c}{\epsilon }_{{\rm{1}}}^{{\rm{c}}}\\ {\epsilon }_{{\rm{2}}}^{{\rm{c}}}\\ {\epsilon }_{{\rm{3}}}^{{\rm{c}}}\end{array}\right)=\left(\begin{array}{ccc}1/\sqrt{3}&1/\sqrt{3}&1/\sqrt{3}\\ 0&-1/\sqrt{2}&1/\sqrt{2}\\ -2/\sqrt{6}&1/\sqrt{6}&1/\sqrt{6}\end{array}\right)\left(\begin{array}{c}{V}_{{\rm{1}}}^{{\rm{c}}}\\ {V}_{{\rm{2}}}^{{\rm{c}}}\\ {V}_{{\rm{3}}}^{{\rm{c}}}\end{array}\right).$$

Physically, ${\epsilon }_{{{1}}}^{\mathrm{c}}$ induces overall gate charge in the qubit-dot array, whereas detuning ${\epsilon }_{{{2}}}^{\mathrm{c}}$ (${\epsilon }_{{\rm{3}}}^{c}$) relocates gate charge within the array along (across) the silicon channel (cf. Fig. 1b). As expected from symmetry, the 111 region within the ${\epsilon }_{{{2}}}^{\mathrm{c}}$–${\epsilon }_{{{3}}}^{\mathrm{c}}$ control plane appears as a triangular region, surrounded by the three two-electron configurations 011, 101, and 110, as indicated by guides to the eye in Fig. 4b. Importantly, due to the finite mutual charging energies within the array (set by interdot capacitances), these three two-electron regions are connected to each other, allowing the cyclic permutation of two electrons without invoking doubly occupied dots (wavefunction overlap) or exchange with a reservoir.

In principle, any closed control loop traversing 011 → 101 → 110 → 011 should exchange the two electrons, which are isolated at all times by Coulomb blockade, making this a topological operation that may find use in permutational quantum computing^26^. In practice, leakage into unwanted qubit configurations (such as 111, 200, 020, etc.) can be avoided by mapping out their ground-state regions, as demonstrated in Fig. 4c by slightly adjusting the operating point ${{V}_{4}^{\mathrm{o}}}$ of the sensor dot. This sensor tuning also allows us to verify the sequence of traversed charge configurations while sweeping gate voltages along the circular shuttling path C, by simultaneously digitizing *V*_H_. The time trace of one shuttling cycle, starting and ending in 011, is plotted in Fig. 4d, and clearly shows the three charge transitions associated with the two-dimensional exchange (i.e., spatial permutation) of two electrons.

## Discussion

In this experiment, only gates G_1_, G_2_, and G_3_ can be pulsed quickly, due to our choice of wirebonding G_4_ as a reflectometry sensor. Therefore, the acquisition in Fig. 4d took much longer (51 s) than the intrinsic speed expected from the characteristic tunneling times in Fig. 3. We did not observe any effects indicating alternative tunneling channels^27^, but verified by intentionally changing the radius of *C* and inspecting *V*_H_(*C*) that leakage into undesired charge configurations does indeed now occur. In future experiments, a faster execution of *C* combined with spin-selective readout^28^ may allow a more direct confirmation of the electrons’ shuttling paths within the array.

We verified that dot 4 can also be depleted to the last electron (see “Methods”) and future work will investigate whether the sensor dot can simultaneously serve as a qubit dot. Our choice of utilizing dot 4 as a charge sensor (read out dispersively from its gate) realizes a compact architecture for spin-qubit implementations where each gate in principle controls one qubit. This technique also alleviates drawbacks associated with the pure dispersive sensing of quantum capacitance, such as tunneling rates constraining the choice of RF carrier frequencies or significantly limiting the visibility of transitions of interest. For example, the honeycomb pattern in Fig. 1d with a clear visibility of dot-4 *and* dot-1 transitions is unusual for gate-based dispersive sensing in the few-electron regime, where small tunneling rates typically limit the visibility of dot-to-lead or interdot transitions^29^. This is a consequence of the strong cross-capacitance between the reflectometry gate G_4_ and dot 1, allowing the RF excitation to probe also the quantum capacitances arising from dot 1. This also explains the visibility of discrete features within the bias triangles of Fig. 1d and shows the potential of gate-based reflectometry for directly revealing excited quantum-dot states. The binary nature of the high-bandwidth charge signal (evident in Fig. 2) may also simplify the algorithmic tuning of qubit arrays^30^.

While all data presented were obtained at zero magnetic field, application of finite magnetic fields to explore spin dynamics and to characterize spin-qubit functionalities should also be possible. In LETI’s silicon-on-insulator technology, coherent spin control was demonstrated for holes in double dots using spin–orbit coupling^10,12^, and electrically driven spin resonance was observed for electrons in double dots using the interplay of spin–orbit coupling and valley mixing^11^. Readout of spin using reflectometry has been demonstrated both for holes^10,12^ and electrons^13,14^.

Another important next step is the application of our findings to larger 2 × *N* devices (see Supplementary Fig. S1 for a 2 × 4 and 2 × 8 device). Unlike linear arrays of qubits^31^, which do not tolerate defective qubit sites, the development of 2 × *N* qubit arrays may prove useful for the realization of fault-tolerant spin-qubit quantum computers, trading topological constraints against lower error thresholds^32^. The systematic loading of such extended arrays with individual electrons, as well as the controlled movement of electrons along the array, can be facilitated by virtual control channels similar to those used in linear arrays^24,33^. Recent experiments even suggest that the capacitive coupling of multiple 2 × *N* arrays on one chip may be possible^34,35^, opening further opportunities for functionalizations and extensions.

Further development of a spin-qubit architecture employing this platform will rely on array initialization^33^, coherent spin manipulation^36^, and high-fidelity operations^37^, as well as readout protocols^14,38^.

In conclusion, we demonstrate a two-dimensional array of quantum dots implemented in a foundry-fabricated silicon nanowire device. Each dot can be depleted to the last electron, and pulsed-gate measurements and single-shot charge readout via gate-based reflectometry allow manipulation of individual electrons within the array, while a common top gate provides an overall tunability of tunnel couplings. We demonstrate that the array is reconfigurable in situ to realize various multi-dot configurations, and utilize the two-dimensional nature of the array to physically permute the position of two electrons. We have also tested device stability, including charge noise (see “Methods” section) and reproducibility upon multiple thermal cycles from room temperature to base temperature (see Supplementary Table S2). In conjunction with complementary experiments in various other laboratories using similar LETI devices from the same fabrication run^14,18,34,35^, these results constitute key steps towards fault-tolerant quantum computing based on scalable, gate-defined quantum dots.

## Methods

### Sample fabrication

Our quantum-dot arrays are fabricated at CEA-LETI using a top-down fabrication process on 300-mm silicon-on-insulator (SOI) wafers, adapted from a commercial fully-depleted SOI (FD-SOI) transistor technology^16^. Compared to single-gate transistors (in which a single-gate electrode wraps across a silicon nanowire) two main changes in regards to gate patterning are needed in order to realize 2 × *N* arrays. First, *N* gate electrodes are patterned, in series along one silicon channel. Second, a dedicated etching process is introduced that creates a narrow trench through the gate electrodes, along the nanowire, thereby splitting each gate electrode into one split-gate pair^17^. The main fabrication steps are described below. For illustrative purposes, the device shown in Fig. 1a was imaged after gate patterning and first spacer deposition^16^, and does not represent the top gate and backend.

Starting with a blank SOI wafer (12 nm Si/145 nm SiO_2_), the active mesa patterning is performed in order to define a thin, undoped nanowire via a combination of deep-ultra-violet (DUV) lithography and chemical etching. The silicon nanowire is 7-nm thin after oxidation, and has a width of ~70 nm for the device studied in this work. Then, a high-quality 6-nm-thick SiO_2_ gate oxide is deposited via thermal oxidation. To define the metal gate, a 5-nm-thick layer of TiN followed by 50 nm of n+-doped polysilicon is used from the standard FD-SOI processing. The gate is patterned using a combination of conventional DUV lithography combined with an electron-beam lithography process, allowing to achieve an aggressive intergate pitch down to 64 nm (gate length, longitudinal gate spacing, and transverse gate spacing as small as 32 nm) without the need for extreme ultraviolet technology. Then, 32-nm-thick SiN spacers between gates and between gates and source/drain regions are formed, which serve two roles: they protect the intergate regions from self-aligned doping (therefore keeping the channel undoped), and they define tunnel barriers within the array. Afterwards, raised source/drain regions are regrown to 18 nm to increase the cross-section of source and drain access. Then, to obtain low access resistances, source/drain are doped in two steps: first with lightly doped drain implant (using As at moderate doping conditions) and consecutive annealing to activate dopants, and then with highly doped drain implant (As and P at heavy doping conditions). To complete the device fabrication, the gate and lead contact surfaces are metallized to form NiPtSi (salicidation), in preparation for metal lines to be routed to bonding pads on the surface of the wafer. Finally, a standard copper-based back-end-of-line process is used to define an optional metallic top gate 300 nm above the nanowire, to make interconnections to bonding pads, as well as to encapsulate the device in a protective glass of silicon oxide. Using the powerful parallelism of foundry fabrication, we obtain dozens of dies on a single 300-mm-diameter wafer, each of them containing hundreds of quantum-dot devices buried 2–3 μm below the chip surface.

### Voltage control

Low-frequency control voltages are generated by a multi-channel digital-to-analog converter (QDevil QDAC) (https://www.qdevil.com), whereas high-frequency control voltages are generated using a Tektronix AWG5014C arbitrary waveform generator. To acquire voltage scans that involve compensated control voltages, we use appropriately programmed QDevil QDACs.

### RF reflectometry

The reflectometry technique is similar to that described in ref. ^19^, in which a sensor dot tunnel-coupled to two reservoirs was monitored via a SMD-based tank circuit wirebonded to the accumulation gate of the sensor. In this work, the sensor dot (located underneath G_4_) is tunnel coupled only to one reservoir (source in Fig. 1a), and the increased cross-capacitance to the three qubit dots results in much larger electrostatic shifts of dot 4 whenever the occupation of the qubit dots changes. For example, each pair of triple points in Fig. 1d is spaced significantly larger than the peak width associated with the sensor-dot transition.

In order to increase the signal intensity as well as to allow for inaccuracies in *α*_4*i*_, we find it useful to occupy the sensor dot with several electrons (6–9 in Fig. 2), and to intentionally power-broaden the Coulomb peaks of dot 4 (with −70 dBm applied to the inductor) for all acquisitions in Fig. 2. The SMD inductance used is 820 nH, and the RF carrier has a frequency of 191.3 MHz. A voltage-controlled phase shifter is used to adjust the phase of the reflected reflectometry carrier relative to the local-oscillator signal powering the mixer. The output of the mixer is low-pass filtered to generate the demodulated voltage *V*_H_. For the data presented here, the phase shifter was adjusted to remove a large background signal in the demodulated voltage, making *V*_H_ sensitive to phase changes in the reflected reflectometry carrier.

For the real-time detection of interdot tunneling events in Fig. 3c, an Alazar digitizing card (ATS9360) is used with a sample rate set to 500 kS/s. The integration time per pixel is set by a 30 kHz low-pass filter (SR560), yielding a signal-to-noise ratio as high as 1.4 in this device.

### Determination of electron number

For a given tuning of the quantum-dot array, the occupation number of each qubit dot is determined by counting the number of discrete electrostatic shifts of the sensor dot (i.e., shifts of a dot-4 Coulomb peak in *V*_H_ along *V*_4_) as the qubit dots are emptied by continuously reducing the control voltage of the dot of interest. If the total number of electrons within the qubit-dot array is desired, voltages *V*_1,2,3_ can be reduced simultaneously, while sweeping *V*_4_ over one or more Coulomb peaks of dot 4, which serves as an electrometer. An example of such a diagnostic scan, for the case of a 111-occupied triple dot, is shown in Supplementary Fig. S3. To determine the number of electrons in the sensor (dot 4), we utilized Coulomb peaks associated with dot 3 as an electrometer for dot 4, while continuously reducing *V*_4_. This works because the strong dispersive signal associated with the dot-3-to-lead transition shows discrete shifts (along *V*_3_) whenever the dot-4 occupation changes (similar to the large mutual shifts evident in Fig. 1d).

### Capacitance matrix

To support our interpretation of dot *i* being localized predominantly underneath gate *i* (*i* = 1, ..., 4), we extract from stability diagrams the capacitances *C*_*ij*_ between gate *j* and dot *i* (in units of aF) for one-electron occupations:

$$\hat{C}=\left(\begin{array}{cccc}2.14&0.33&0.25&0.73\\ 0.3&1.69&0.22&0.17\\ 0.32&0.6&1.41&0.26\\ 0.79&0.34&0.47&2.00\end{array}\right).$$

In this capacitance matrix, the relatively large diagonal elements reflect the strong coupling between each gate and the dot located underneath it. By adding several electrons to the array, we have also observed that the capacitances change somewhat, indicating a spatial change of wavefunctions (not shown) and suggesting an alternative way to change tunnel couplings.

### Fitting tunneling times

In Fig. 3c we show 100 single-shot traces (upper panel) and the average of all traces. The average has been fitted by an exponential decay with the initial value, the 1/*e* time, and the long-time limit (offset) as free fit parameters. For plotting purposes, ${\bar{V}}_{{\rm{H}}}$ is then calculated by substracting the offset from the average, and dividing the result by the initial value. For clarity of presentation (the sampling rate for raw data of Fig. 3c was 500 kS/s), in the lower panel of Fig. 3c we also decimated the time bins by a factor of 4. Such a decimation was also used for plotting the data related to the other transitions investigated, as reported in Supplementary Fig. S2.

### Assessing device stability

At base temperature of our dilution refrigerator (≲50 mK) the charge noise of the device is estimated as follows. The device is configured as a single quantum dot and the current flow is measured in the presence of a small source–drain voltage. Due to Coulomb blockade, current peaks as a function of gate voltage can then be used to measure the effective gate-voltage noise, by measuring the noise spectrum of the current and converting it to gate-voltage noise based on the first derivative of current with respect to gate voltage^18^. Using the gate-voltage lever arm, we convert the inferred gate-voltage noise into an effective noise in the chemical potential of the quantum dot, yielding  ~1.1 μeV/$\sqrt{\mathrm{Hz}}$ at 1 Hz. This value should be regarded as an upper bound (as it does not take instrumentation noise into account), and is comparable to the best values we found in literature for Si/SiGe-based quantum dots^39^.

In addition to charge noise, we report the spread in gate voltages needed to accumulate the first electron in each dot (which we refer to as threshold voltage), and their reproducibility in different cool downs. When measuring the three double dots in Fig. 2a–c, the non-participating gate voltages (*V*_3_, *V*_1_, and *V*_2_, respectively) are fixed at zero. Therefore, the position and size of the shown Coulomb diamonds represent the variation of threshold voltages within this array. The sloped boundaries arise from capacitive cross coupling (off-diagonal elements of $\hat{C}$), and imply that the voltage threshold for the 0-to-1 transition of a particular gate electrode depends on the values of the other gate voltages. To facilitate comparison of 0-to-1 threshold voltages between different gate electrodes (and between different cool downs), the observed slope of a particular charge transition in the five-dimensional gate-voltage space can be used to extrapolate from the observed threshold voltage of each gate electrode to a hypothetical gate-voltage configuration where all other side gates are held at zero volt. Threshold voltages from three different cool downs of the same device are provided in Supplementary Table S2. The observed spread in extrapolated threshold voltages for different gate electrodes (of order 40 mV) is comparable to the change of voltage thresholds when warming the device to room temperature and cooling it back down, consistent with homogeneous gate definition during fabrication.

### Reporting summary

Further information on research design is available in the Nature Research Reporting Summary linked to this article.

## Supplementary information

Supplementary Information

Reporting Summary

## Data Availability

The datasets generated and analyzed during the current study are available from the corresponding author (F.K.) upon reasonable request.
