# Supplementary material for: Single-electron operations in a foundry-fabricated array of quantum dots
Source: Nat Commun. 2020 Dec 16;11:6399. doi: 10.1038/s41467-020-20280-3 (PMC7744547; doi:10.1038/s41467-020-20280-3)
Supplement: Supplementary file 1 — Supplementary Information [file 41467_2020_20280_MOESM1_ESM.pdf]

Supplementary Information for
"Single-electron operations in a foundry-fabricated array of quantum dots"

Fabio Ansaloni†,[1] Anasua Chatterjee†,[1] Heorhii Bohuslavskyi,[1]
Benoit Bertrand,[2] Louis Hutin,[2] Maud Vinet,[2] and Ferdinand Kuemmeth[1,3]

[1] *Center for Quantum Devices, Niels Bohr Institute, University of Copenhagen, 2100 Copenhagen, Denmark*
[2] *CEA, LETI, Minatec Campus, Grenoble, France*
[3] *kuemmeth@nbi.dk*
† *These authors contributed equally to this work.*

(Dated: December 4, 2020)

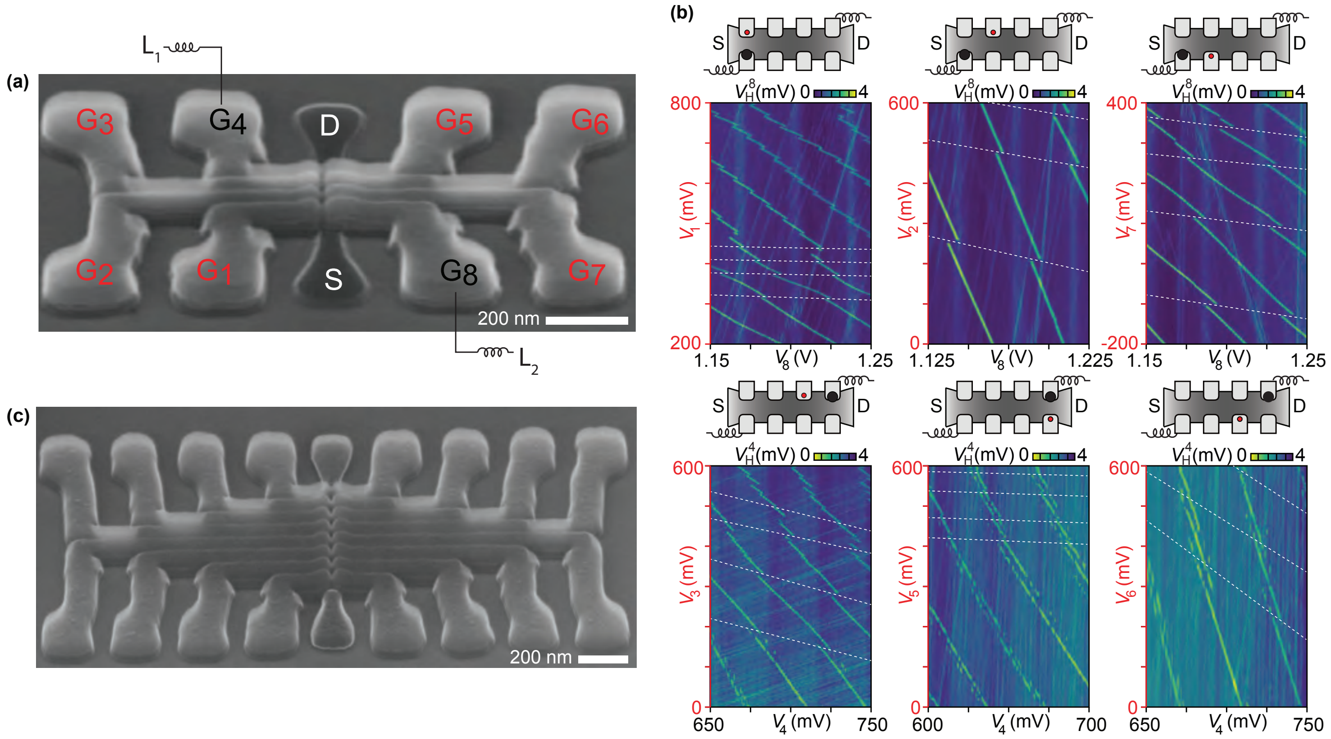

FIG. S1: **2xN quantum-dot arrays.** (a) Tilted scanning-electron micrograph of a 2x4 quantum-dot array. Ohmic contacts appear dark grey and are used as electron reservoirs. Polysilicon electrodes appear light grey and are used to control the chemical potential of each quantum dot. Gates G_4 and G_8 are embedded in high-frequency reflectometry circuits to perform gate-based dispersive readout, analogously to Figure 1b,d, resulting in homodyne-detected voltages $V_{\rm H}^4$ and $V_{\rm H}^8$, respectively. (b) Detection of the first electron for each of the quantum dots in the array. A multielectron quantum dot formed underneath G_8 is used as a sensor to investigate the left side of quantum dot array (top graphs), whereas the right side is investigated using G_4 as sensor dot (bottom graphs). In the insets, red and black dots denote one- and multi-electron occupation, respectively. (c) Tilted scanning-electron micrograph of a 2x8 quantum-dot array.

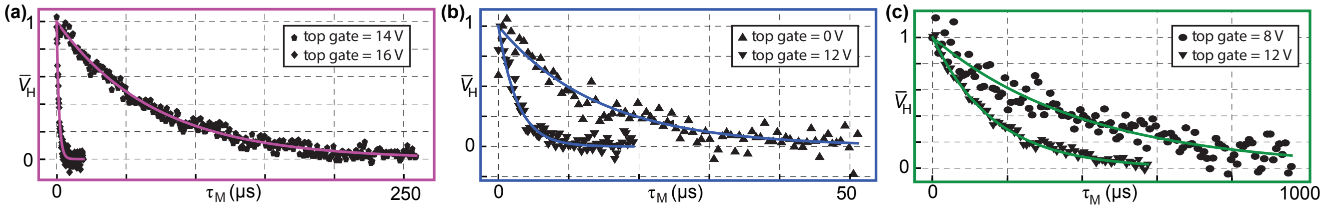

FIG. S2: **Sensor signal and fitted tunneling times for the other transitions in Figure 3a.** (a)-(c) An exponential decay is fitted to the normalized $\bar{V}_{\mathrm{H}}$ data for different values of the top-gate voltage, analogous to the interdot transition described in Figure 3c. Fitted $1/e$ times are plotted in Figure 3d. Colors correspond to the transitions indicated in Figure 3a.

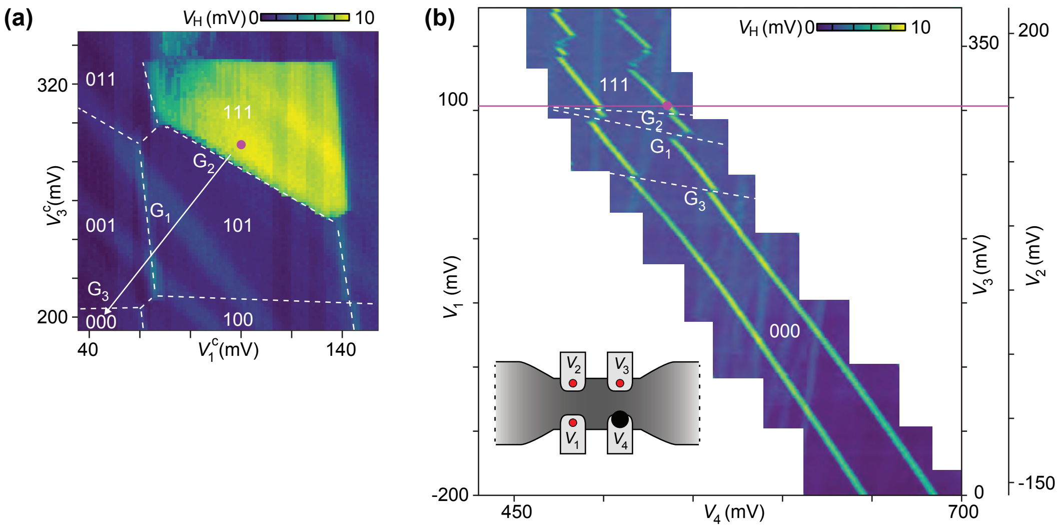

FIG. S3: **Verifying electron count in the 111 configuration.** (a) Putative 111 ground-state region (yellow area) appearing in a compensated 2D map (V_1^c vs V_3^c) similar to the maps in Figure 2. The qubit array, initialized at the magenta dot, can be depleted of electrons by reducing simultaneously V_1^c and V_3^c (white arrow), in this example removing one electron from dot 2, then one from dot 1, and finally one from dot 3 (ground-state transitions are marked by G_2, G_1, G_3). However, these charge transitions are not visible in the sensor signal V_{H}, as dot 4 is in Coulomb blockade (blue regions), thereby neither confirming nor disputing the absolute occupation numbers. (b) However, by reducing V_1, V_2, V_3 simultaneously (note the three vertical axes) while sweeping V_4, each charge transition within the qubit array induces a capacitive shift of the dot-4 Coulomb peak (magenta dot), confirming the presence of exactly three electrons in the 111 configuration. Moreover, comparison with another dot-4 Coulomb peak reveals three distinct slopes (marked G_2, G_1, G_3), confirming that each charge transition indeed corresponds to a different dot. Note the absence of charge transitions for lower or even negative gate voltages applied to V_1, V_2, and V_3.

V_1 (mV)	V_2 (mV)	V_3 (mV)	V_4 (mV)	V_{tg} (mV)
dot2-to-lead transition				
0	131	112	606	8000
0	91	98	500	10000
0	51	53	510	12000
dot3-to-lead transition				
0	195	251	615	0
0	135	204	633	2000
0	65	166	635	4000
0	40	144	615	6000
0	35	102	626	8000
0	5	83	565	10000
0	-30	62	500	12000
dot1-to-dot2 transition				
98	210	0	480	6000
63	148	0	438	8000
17	122	0	541	10000
dot2-to-dot3 transition				
0	100	113	551	10000
0	51	107	343	14000
0	-1	66	306	16000

TABLE S1: **Gate voltages associated with Figure 3d.** Each data point in Figure 3d shows the observed tunneling time associated with a specific charge transition, specified by the color code in Figure 3a. The gate voltages associated with each charge transition are listed here. For each transition, the side-gate voltages needed to be decreased somewhat when increasing the top-gate voltage (V_{tg}), due to capacitive cross coupling between gate electrodes and quantum dots.

cool down		observed 0-1 threshold V_1, V_2, V_3, V_4, V_{tg} (mV)	extrapolated 0-1 threshold V_1, V_2, V_3, V_4, V_{tg} (mV)
A	dot-1	**44**, 80, 150, 491, 12000	**292** , 0, 0, 0, 10000
	dot-2	80, **40**, 150, 489, 12000	0, **248** , 0, 0, 10000
	dot-3	80, 100, **95**, 489, 12000	0, 0, **280**, 0, 10000
B	dot-1	**65**, 134, 170, 510, 10000	**328**, 0, 0, 0, 10000
	dot-2	101, **85**, 170, 505, 10000	0, **258**, 0, 0, 10000
	dot-3	101, 120, **133**, 519, 10000	0, 0, **282**, 0, 10000
C	dot-1	**60**, 111, 175, 508, 12000	**353**, 0, 0, 0, 10000
	dot-2	90, **67**, 175, 508, 12000	0, **274**, 0, 0, 10000
	dot-3	90, 119, **132**, 510, 12000	0, 0, **300**, 0, 10000

TABLE S2: **Threshold voltages for different gate electrodes and different cool downs.** Comparison between other cool downs of the same device as in Figure 2, listing gate voltages for which the 0-to-1 charge transition was observed for each qubit dot ("observed threshold"). Voltages associated with the strongest gate electrode for a given dot (cf. diagonal entries of $\hat{C}$ in the main text) are printed in bold face. To facilitate comparison between different cool downs, as well as comparison between different gate electrodes within one cool down, we use lever arms associated with $\hat{C}$ to extrapolate from the observed threshold a fictive threshold that corresponds to biasing a single gate electrode while keeping all other gate electrodes at zero (rightmost column). The spread of extrapolated threshold voltages for cool down A (B, C) is 44 (54, 47) mV, whereas the spread of extrapolated threshold voltages between different cool downs is 59 (26, 20) mV for gate 1 (2, 3). For each cool down, gate electrodes were grounded while cooling from room temperature to base temperature of the dilution refrigerator.
